# Dynamics of hormonal disorders following unilateral orchiectomy for a testicular tumor

**DOI:** 10.1007/s12032-017-0943-0

**Published:** 2017-04-07

**Authors:** Paweł J. Wiechno, Maria Kowalska, Jakub Kucharz, Małgorzata Sadowska, Wojciech Michalski, Grażyna Poniatowska, Joanna Jońska-Gmyrek, Joanna Rzymkowska, Karol Nietupski, Tomasz Demkow

**Affiliations:** 1 Department of Uro-Oncology, Maria Sklodowska-Curie Memorial Cancer Center and Institute of Oncology, Roentgena 5 st., 02-781 Warsaw, Poland; 2 Laboratory of Tumor Markers, Department of Pathology and Laboratory Diagnostics, Maria Sklodowska-Curie Memorial Cancer Center and Institute of Oncology, Roentgena 5 st., 02-781 Warsaw, Poland; 3 Department of Experimental and Clinical Surgery, Jagiellonian University Medical College, Michalowskiego 12 st., Cracow, Poland

**Keywords:** Testicular tumor, Orchiectomy, Testosterone, Hormones

## Abstract

Testicular tumors and their treatment interfere with homeostasis, hormonal status included. The aim of the study was to evaluate hormonal disorders of the pituitary–gonadal axis in men treated for testicular tumors. One hundred twenty-eight men treated for a unilateral testicular tumor at our institution were included. The hormonal status was prospectively evaluated in 62 patients before orchiectomy, 120 patients 1 month after orchiectomy and 110 patients at least 1 year after the treatment. The concentrations of human chorionic gonadotropin (hCG), testosterone (T), estradiol, luteinizing hormone (LH), follicle-stimulating hormone (FSH) and prolactin were measured. The clinically significant testosterone deficiency was defined either as testosterone <2.31 ng/mL or testosterone within the range of 2.31–3.46 ng/mL but simultaneous with T/LH ratio ≤1. Changes in hormone levels were significant: LH and FSH rose in the course of observation, and the concentration of hCG, testosterone, estradiol decreased. PRL concentration was the lowest at 1 month after orchiectomy. In multivariate analysis, the risk of the clinically significant testosterone deficiency was 0.2107 (95% CI 0.1206–0.3419) prior to orchiectomy, 0.3894 (95% CI 0.2983–0.4889) 1 month after surgery and 0.4972 (95% CI 0.3951–0.5995) 1 year after the treatment. The estradiol concentration was elevated in 40% of patients with recently diagnosed testicular cancer and that was correlated with a higher risk of testosterone deficiency after the treatment completion. Hormonal disorders of the pituitary–gonadal axis in men treated for testicular tumors are frequent. The malignant tissue triggers paraneoplastic disorders that additionally disturb the hormonal equilibrium.

## Introduction

Affecting mostly Caucasian males aged 15–40, testicular germ cell tumors represent the most common malignancy in this age group. Presenting symptoms usually include a testicular mass as well as lesions along the body mid-line (i.e., retroperitoneum, mediastinum or brain). Clinically divided into seminomas and non-seminomas, each group accounting for approximately 50%, they are a fine example of success in oncology. The prognosis remains excellent, especially in testicle-limited (stage I) tumors. Overall survival rates reach 99% for stage I seminomas. On the other hand, metastatic or relapsed disease does not preclude radical approach and chances for cure. Even in the ‘poor prognosis’ group of metastatic non-seminoma, 48–60% can still be cured with first-line chemotherapy [1]. Also older patients (>65 years) with germ cell tumors, believed to have a worse prognosis, achieve a survival rate as high as 72–83% [2]. Often combined with surgery and radiotherapy, chemotherapy has been the cornerstone of the treatment. New approaches such as high-dose chemotherapy with autologous stem cell transplant are being investigated to increase the survival rates in poor prognosis patients. Nevertheless, the clinical importance has now shifted from pursuing optimal treatment methods to closer follow-up of cancer survivors and an early diagnosis of late toxicities. As many as 24% of testicular cancer survivors develop overweight, 24%—hypercholesterolemia—and 30%—hypertension. Survivors with testosterone levels <4.3 ng/mL (22%) have an increased risk of the metabolic syndrome [3]. This effect is now attributed to the cumulative dose of cisplatin [4]. Uncompensated hypogonadism is characterized by the testosterone concentration below the lower limit of normal. In compensated hypogonadism, the testosterone concentration is within normal range, while the LH concentration exceeds the upper limit of normal. Impaired post-pubertal androgen function may cause infertility, sexual disorders, muscle weakness and bone demineralisation, as well as other metabolic disorders, depression and cognitive impairment [5, 6]. Male hypogonadism is defined as a syndrome of clinical symptoms resulting from androgen deficiency. It stems either from impaired function of the gonads (primary hypogonadism) or from hypothalamic–pituitary disorders at different levels (secondary hypogonadism). The most common causes of the primary hypogonadism are Klinefelter syndrome and testicular tumors [7–9]. The diagnosis of clinically significant testosterone (T) deficiency based solely on the total testosterone concentration is problematic. Total testosterone must exceed 3.50–4.00 ng/dL to reliably predict normal free testosterone [10]. According to the recommendations published in 2009 to be used in clinical practice, men with total serum of testosterone <2.31 ng/mL (8 mmol/L) should be treated with hormone replacement therapy. For men with total testosterone values between 2.31 and 3.46 ng/mL (8 and 12 mmol/L), treatment should be considered in the presence of symptoms associated with testosterone deficiency [11]. T (ng/mL)/LH (mIU/mL) ratio can facilitate the decision in men with borderline testosterone. The ratio reflects the complex nature of testosterone deficiency in adulthood that originates from hypothalamic–pituitary–gonadal axis disturbances. The T/LH ratio ≤1 correlates with the presence of testosterone deficiency symptoms [12]. Knowledge of hormonal disorders in patients treated for testicular tumor will allow to predict and possibly avoid metabolic consequences of hypogonadism. The aim of the paper is to evaluate the dynamics of hormonal changes in the pituitary–gonadal axis in adult men treated for testicular tumors.

## Materials and methods

One hundred twenty-eight patients with a unilateral testicular tumor were included in the study. The procedures had been approved by the Ethical Committee of Maria Sklodowska-Curie Memorial Cancer Center and Institute of Oncology, Warsaw, Poland.

The study was prospective, non-interventional. Written informed consent was obtained from each patient.

The study procedures were carried out at three control points:

- directly before orchiectomy (if the patient was referred prior to surgery),
- one month after orchiectomy,
- at least 1 year after the completion of treatment. Patients with progressive disease after the first-line treatment were excluded from the analysis at control points 2 and 3.

The data were collected at Maria Sklodowska-Curie Memorial Cancer Center and Institute of Oncology, Warsaw, Poland, between 2009 and 2013. Taking into account the patients lost to follow-up, control point procedures were conducted 292 times: 62 times for the first control point, 120 for the second and 110 for the third one. In multivariate analyses, five stage I patients progressing after the completion of the second control point procedures were excluded due to possible disturbing effect of the recurrence. Characteristics of the clinical data are presented in Table 1. One month after orchiectomy, the patients were divided into two groups: with or without active disease. At control points, the patients had their blood drawn during routine visits. The plan was to assess the pituitary–gonadal axis hormones, i.e., LH (normal values: 1.7–8.6 mIU/mL), FSH (normal values: 1.5–12.4 mIU/mL), testosterone (normal values: 3.47–8 ng/mL), as well as those potentially affecting the axis, i.e., estradiol (normal values: 7.6–42.6 pg/mL), prolactin (normal values: 4.6–21.4 ng/mL) and beta-hCG (normal values: <5 mIU/mL), testosterone (normal values: 3.47–8.0 ng/mL). It was decided to recognize testosterone deficiency at values <2.31 ng/mL (8 mmol/l). The gray zone consisted of patients with testosterone values of 2.31–3.46 ng/ml (8–12 mmol/l). For the gray zone, the clinically significant testosterone deficiency was diagnosed if the T/LH ratio was ≤1. Changes in the hormone concentrations were analyzed at the above-mentioned control points. The reactivity of Leydig cells to stimuli from the pituitary gland was evaluated on the basis of T/LH ratio. Associations between hormone concentrations at the planned control points were analyzed. We used the general linear model (GLM) and logarithmic transformation to evaluate changes in hormone concentrations. In the multivariate generalized linear mixed models, data are organized according to the random factors. The mixed effect models could therefore recover information in the data not found by the traditional methods, especially for incomplete or unbalanced data. Percentage changes were addressed by using a log-transformation in the models as a link functions. To overcome the problem with outliers the gamma distribution was used. Log scale in the pictures is a consequence of log links introduced in the models. Dichotomous parameters were analyzed by the ‘logit’ linking function. Moreover, the models comprised confounding factors, e.g., age and clinical stage. The calculations were carried out with GLIMMIX procedure in the SAS software (version 12.3). In the analysis of hormone levels, we used Pearson’s linear correlation coefficient. The correlation was considered strong with coefficient values >0.5 for positive correlations and <−0.5 for negative correlations. The results were considered statistically significant at *p* < 0.05.

**Table 1:** Characteristics of the study group

Participants	128 men
Patients’ age (median)	18–63 years (31 years)
Analyzed	
First control point	62 patients
Second control point	120 patients
Third control point	110 patients
Histology of the primary testicular tumor	
Seminoma	59 patients
Non-seminoma	67 patients
*Seminoma* component present	14 patients
*Ca. embryonale* component present	56 patients
*Choriocarcinoma* component present	12 patients
*Yolk sac tumor* component present	27 patients
*Teratoma immaturum* component present	12 patients
*Teratoma maturum* component present	22 patients
Leydig cell tumor	2 patients
Clinical stage	
IA	59 patients
IB	15 patients
IS	5 patients
IIA	16 patients
IIB	2 patients
IIC	2 patients
IIIA	13 patients
IIIB	8 patients
IIIC	8 patients
Treatment methods	
Unilateral orchiectomy	128 patients
Chemotherapy	70 patients
Radiotherapy	16 patients
Retroperitoneal lymph node dissection	11 patients

## Results

### Changes in hormone concentrations over time

**Fig. 1:**
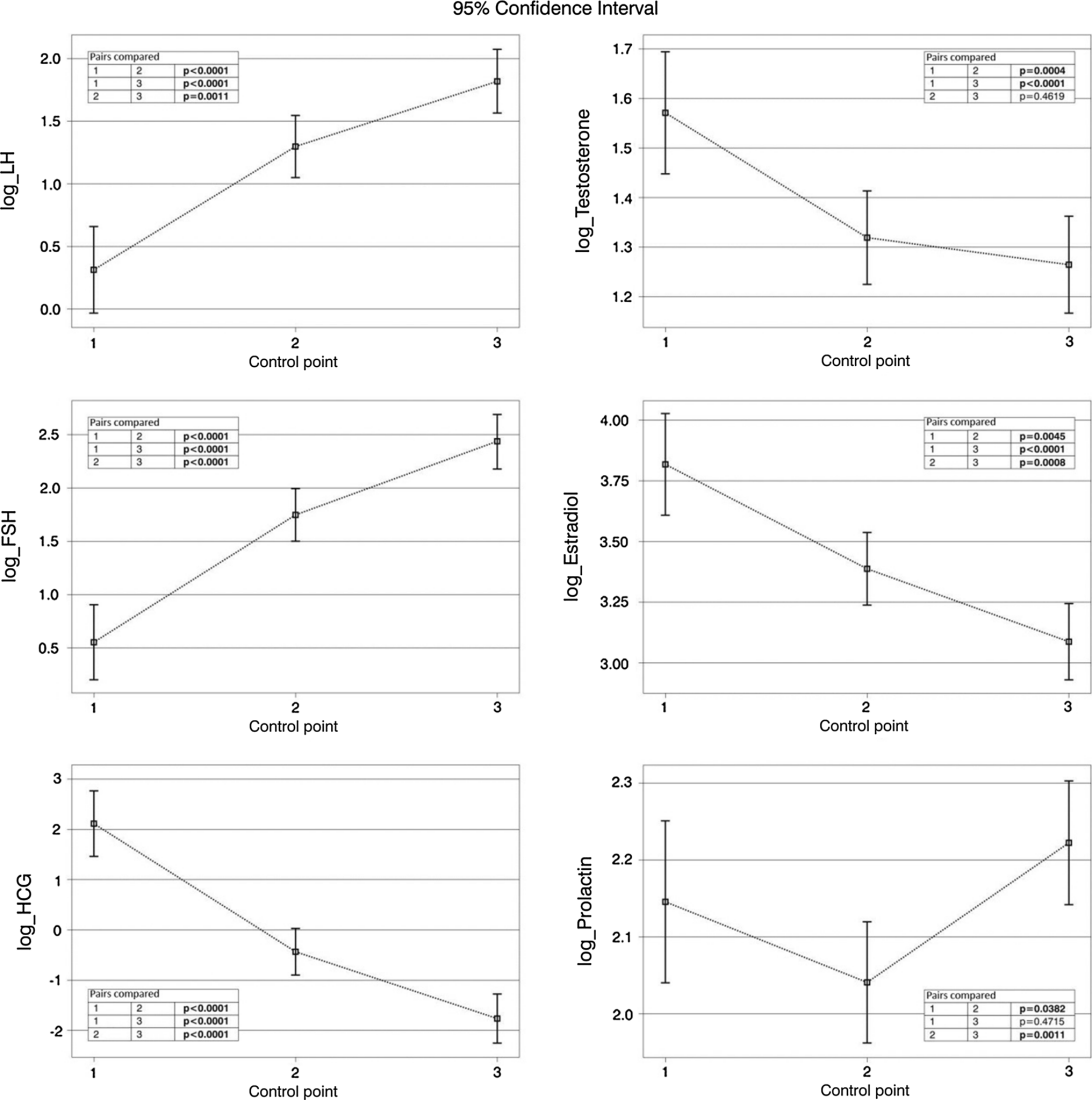
Changes in hormone concentrations at control points (logarithmic transformation)

The concentrations of both pituitary gonadotropins, LH and FSH, increased significantly at subsequent control points, not only directly after orchiectomy. In the course of treatment, the concentration of tumor gonadotropin, hCG, decreased and returned to normal in all patients within 1 year after the completion of treatment. We observed a significant decrease in testosterone concentration directly after orchiectomy. One year after the treatment, testosterone concentrations still remained significantly lower than before orchiectomy. 40% of patients with active disease presented with pathologically high estradiol concentrations, exceeding the normal range even 50-fold. However, the estrogen concentrations were decreasing significantly during treatment. Additionally, a statistically significant decrease in prolactin concentrations was observed directly after orchiectomy. Similarly, these values returned to normal 1 year after completion of the anticancer treatment. Table 2 shows concentrations of the hormones analyzed at a given control point. Figure 1 shows these results graphically.

**Table 2:** Hormone concentrations at control points

Control point	Minimum	Maximum	Median	Number of patients with values above normal limits	Number of patients with values below normal limits
Chorionic gonadotropin (mIU/mL)					
1.	0	533.390	1.95	26/62 (42%)	0
2.	0.1	549.893	0.1	23/120 (19%)	0
3.	0.1	4.1	0.1	0/110	0
Prolactin (ng/mL)					
1.	3.80	34.60	8.55	2/58 (4%)	2/58 (3%)
2.	2.80	21.90	7.6	1/117 (1%)	15/117 (13%)
3.	3.55	21.1	9.7	0/110	4/110 (3%)
Estradiol (pg/mL)					
1.	5.00	2228.00	34.2	22/57 (39%)	1/57 (2%)
2.	4.00	2333.00	27.00	18/115 (16%)	2/115 (2%)
3.	5.00	101.70	22.91	7/110 (6%)	9/110 (8%)
Follicle-stimulating hormone (mIU/mL)					
1.	0.10	29.40	4.96	6/58 (10%)	22/58 (38%)
2.	0.10	58.40	11.1	37/120 (31%)	18/120 (15%)
3.	0.20	66.31	16.79	55/110 (50%)	3/110 (3%)
Luteinizing hormone (mIU/mL)					
1.	0.10	13.70	2.9	5/58 (9%)	23/58 (40%)
2.	0.10	27.20	6.51	/120 (%)	18/120 (15%)
3.	0.10	135.00	7.01	43/110 (39%)	4/110 (4%)
Testosterone (ng/mL)					
1.	1.10	15.00	5.05	12/58 (21%)	<2.31: 3/58 (5%) ≥2.31 and ≤3.46: 10/58 (17%)
2.	0.60	10.30	3.8	6/120 (5%)	<2.31: 19/120 (16%) ≥2.31 and ≤3.46: 32/120 (27%)
3.	1.30	12.00	3.59	2/110 (2%)	<2.31: 19/110 (17%) ≥2.31 and ≤3.46: 33/110 (30%)

### The risk of testosterone deficiency

**Fig. 2:**
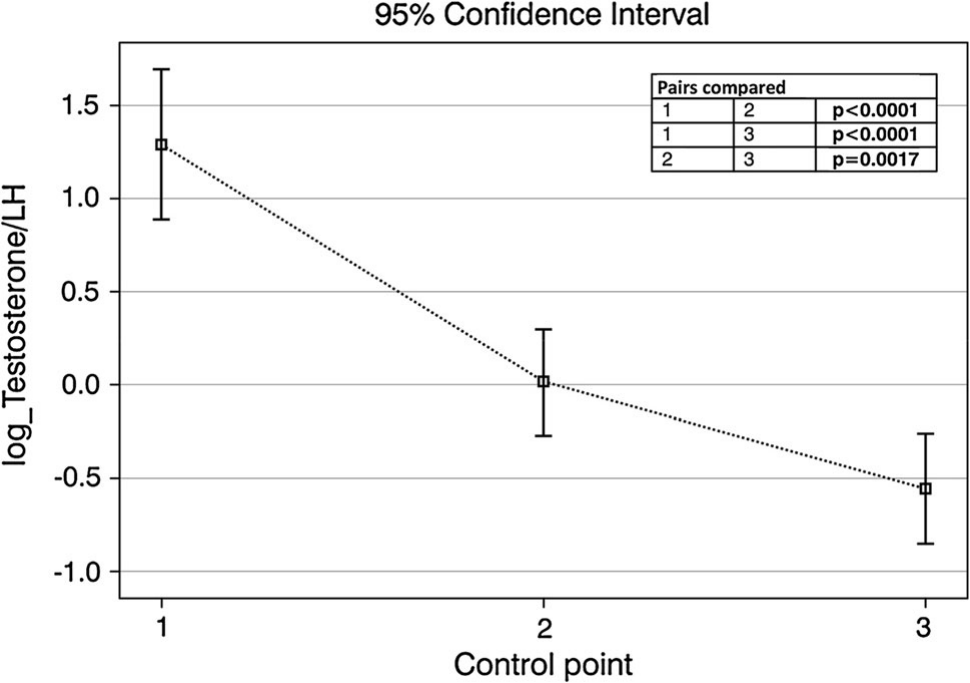
Testosterone/LH ratio at control points

In the presented group of patients, testosterone concentration below 2.31 ng/mL was found in 5% of patients prior to orchiectomy, 16% of patients a month after orchiectomy and 17% of patients at least 1 year after curative treatment. The values of testosterone between 2.31 and 3.46 ng/mL were detected in 17% of patients at the first control point, 27% of patients in the second and 30% of patients in the third control point. After taking into account the T/LH ratio ≤1, the clinically significant testosterone deficiency was diagnosed at three successive checkpoints in additional 12, 23 and 29% of patients. In the multivariate analysis, taking into account the clinical stage and the patient’s age, it was found that the risk of clinically relevant testosterone deficiency was 0.2107 before orchiectomy (95% CI 0.1206–0.3419), 0.3894 one month after orchiectomy (95% CI 0.2983–0.4889) and 0.4972 one year after curative treatment (95% CI 0.3951–0.5995). The risk of clinically significant testosterone deficiency at the second and the third control points was higher than in the first one. That difference was statistically significant (*p* = 0.0121 and *p* = 0.0005, respectively). Patients treated for testicular tumors proved to present with lowered testosterone/LH ratio, which was aggravating over time and statistically significant—Fig. 2.

### The correlation between hormone concentrations

A strong negative correlation was found between beta-hCG concentration and both pituitary gonadotropins in patients with active disease. In these patients, estradiol concentration was negatively correlated with LH and FSH concentrations, whereas beta-hCG was positively correlated with testosterone (before orchiectomy) and prolactin concentrations. These associations were statistically significant. Estradiol concentrations correlated positively with prolactin concentrations in this group of patients. Such correlations are not present in the group of patients free of disease. The findings are shown in Table 3. In multivariate analysis, taking into account patients age, clinical stage as well as the concentrations of chorionic gonadotropin and estradiol a month after orchiectomy, it was demonstrated that elevated estradiol predicted clinically relevant testosterone deficiency (*p* = 0.0288) after treatment completion. The effect of high concentrations of beta-hCG was not statistically significant (*p* = 0.0777).

**Table 3:** Correlations between concentrations of hormones associated with the pituitary–gonadal axis

	LH	FSH	Testosterone	Estradiol	HCG	PRL
*Active disease*						
First control point—before orchiectomy						
LH	1.0	**0.92393**	**−0.63824**	**−0.64714**	**−0.67406**	**−**0.36690
FSH	**0.92393**	1.0	**−0.64079**	**−0.79251**	**−0.72811**	**−**0.48402
Testosterone	**−0.63824**	**−0.64079**	1.0	**0.63215**	**0.62279**	0.40277
Estradiol	**−0.64714**	**−0.79251**	**0.63215**	1.0	**0.84003**	**0.66343**
HCG	**−0.67406**	**−0.72811**	**0.62279**	**0.84003**	1.0	**0.56137**
PRL	**−**0.36690	**−**0.48402	0.40277	**0.66343**	**0.56137**	1.0
Second control point—1 month after orchiectomy—patients with active disease						
LH	1.0	**0.93580**	**−**0.25795	**−0.51236**	**−0.65898**	**−**0.28004
FSH	**0.93580**	1.0	**−**0.38360	**−0.63227**	**−0.75261**	**−**0.30979
Testosterone	**−**0.25795	**−**0.38360	1.0	**0.61473**	0.35639	0.30749
Estradiol	**−0.51236**	**−0.63227**	**0.61473**	1.0	**0.68578**	0.30295
HCG	**−0.65898**	**−0.75261**	0.35639	**0.68578**	1.0	0.25110
PRL	**−**0.28004	**−**0.30979	0.30749	**0.69001**	0.25110	1.0
*Free of disease*						
Second control point—1 month after orchiectomy—patients free of disease						
LH	1.0	**0.83210**	**−**0.09518	**−**0.31178	**−**0.47824	**−**0.05733
FSH	**0.83210**	1.0	**−**0.13473	**−**0.30350	**−**0.47802	**−**0.10608
Testosterone	**−**0.09518	**−**0.13473	1.0	0.24555	0.12947	0.03140
Estradiol	**−**0.31178	**−**0.30350	0.24555	1.0	0.42551	0.14004
HCG	**−**0.47824	**−**0.47802	0.12947	0.42551	1.0	0.22823
PRL	**−**0.05733	**−**0.10608	0.03140	0.14004	0.22823	1.0
Third control point—1 year after treatment completion						
LH	1.0	**0.83956**	**−**0.12871	**−**0.19528	0.06883	0.08902
FSH	**0.83956**	1.0	**−**0.18038	**−**0.21381	0.09891	0.04118
Testosterone	**−**0.12871	**−**0.18038	1.0	0.52583	**−**0.11595	0.24094
Estradiol	**−**0.19528	**−**0.21381	0.52583	1.0	**−**0.20111	0.07692
HCG	0.06883	0.09891	**−**0.11595	**−**0.20111	1.0	0.14737
PRL	0.08902	0.04118	0.24094	0.07692	0.14737	1.0

Bold font indicates strong and very strong correlations, for all *p* < 0.0001

## Discussion

It has been estimated that the annual decrease in the circulating testosterone concentration is 0.2–2.0%. The percentage of middle-aged men presenting with hypogonadism is 6% [13]. In comparison, patients treated for a testicular malignancy performed worse. According to the literature, the risk of decreased testosterone concentration many years after a successful treatment of testicular tumors is 5–25% [8, 9, 14–20]. In contrast, it has also been reported that 10 years after orchiectomy testosterone concentrations do not differ from those in control groups [8]. All authors [8, 9, 14–20] agree that chronic compensated hypogonadism continues to be an issue even over 10 years after the completion of treatment [8]. According to other investigators, 24–75% of patients present with abnormal LH concentrations many years after the anticancer treatment. In addition, the upper range of the values given above seems to be better documented [16, 18–20]. In the presented material, the risk of pituitary-Leydig cell axis insufficiency increased over time, even after the completion of treatment. Moreover, the LH concentration seems to better reflect hormonal changes in men years after unilateral orchiectomy [8, 21]. The lowered testosterone/LH concentration ratio directly after orchiectomy may be a sign of a decrease in volume of the testosterone-releasing tissue after an LH stimulus. However, the subsequent decrease in this ratio should rather be interpreted as a decreased reactivity of Leydig cells in response to LH stimulation. This finding is confirmed by other authors [8, 21]. Luteinizing hormone and chorionic gonadotropin are morphologically similar polypeptide hormones stimulating the same receptor [22]. This fact explains the disruptive effect of high beta-hCG concentrations on the pituitary–gonadal axis. Other authors also suggest that hCG concentrations in testicular cancer patients correlate with testosterone, prolactin, estradiol and gonadotropins concentrations [23–25]. In our study, nearly 40% of the patients with active disease presented with elevated estradiol concentrations. In contrast, estradiol concentrations exceeded the normal limits in only 7% of patients without active disease. In the former group, we found a very strong correlation between the elevated estradiol concentrations and high beta-hCG concentrations. This justifies the statement that estradiol fulfills the criteria of a serum marker of the malignancy. According to other authors, an increasing estradiol concentration may predict a recurrence, even when other tumor markers remain within normal limits [26]. However, the role of estradiol as a tumor marker is still undetermined. Pathologically high estradiol concentrations interfere with the pituitary–gonadal axis, as confirmed by other authors [27, 28]. In the presented group of patients, abnormally high estradiol concentrations in the course of the disease were shown to have long-term consequences, increasing the risk of testosterone deficiency after treatment completion. The source of such high estradiol concentrations in patients with testicular tumors is unclear. There have been scarce data that estradiol may be secreted directly by the tumor, when exposed to chorionic gonadotropin [29, 30]. Another potential source of estradiol may be Leydig cells, stimulated by high hCG concentrations [30].

In our material, prolactin concentrations also changed significantly over time; this leads to a conclusion that prolactin balance is disrupted in patients with testicular tumors. Moreover, the balance is strongly dependent on hCG and estradiol concentrations, which has been confirmed in other papers [31]. The changes in hormone concentrations cannot be attributed only to unilateral orchiectomy. The clinical picture suggests that the tumor tissue also plays an important role in the endocrine balance through the paraneoplastic mechanism. Hypogonadism is a common problem in testicular cancer survivors. Yet, the need and principles of hormone supplementation are still to be established in further studies.

The study was limited by the fact that some of the patients were lost to follow-up. Similarly, the small number of patients included prior to orchiectomy could reduce the study’s power.

## Conclusions

Changes in hormone concentrations in men treated for a unilateral testicular tumor are significant: LH and FSH concentrations increase in the course of treatment, while the concentrations of hCG, testosterone, estradiol decrease. Prolactin is the lowest at 1 month after orchiectomy. Pathologically high concentrations of chorionic gonadotropin and estradiol in these patients interfere with the pituitary–gonadal axis in a paraneoplastic mode.
